# Operationalizing a walking exercise prescription based on 6-minute walk test results

**DOI:** 10.21203/rs.3.rs-9926912/v1

**Published:** 2026-06-22

**Authors:** Josh Sepic, Pamela M Bloomer, Susanna Miranda, Christopher B Hughes, Fei-Pi Lin, Andres Duarte-Rojo

**Affiliations:** Northwestern Feinberg School of Medicine & Shirley Ryan Ability Lab; University of Pittsburgh Medical Center; Northwestern Medicine; University of Pittsburgh Medical Center; University of Pittsburgh Medical Center; Northwestern Medicine

**Keywords:** frailty, sarcopenia, fitness, walking, exercise

## Abstract

A walking prescription is both practical and effective to prepare patients for liver transplant. To guide healthcare professionals on the number of steps to be prescribed, we correlated 6-minute walk test results with average daily collected with a personal fitness tracker. Based on our results, we recommend a 1500, 3000, and 6000 steps/day prescription for candidates strolling < 250 m, 250–349 m, and ≥ 350 m on their 6-minute walk test, respectively.

## Introduction

Providing a tailored exercise prescription to liver transplant (LT) candidates can be challenging^(1)^. Walking is a proven exercise intervention for improving physical fitness assessed via the 6-minute walk test (6MWT) and the liver frailty index (LFI) in LT candidates^(2)^. However, access to supervision can become a limiting factor in participation for many. Physical activity trackers (PAT) provide an opportunity to monitor and prescribe exercise, remotely. To date, several studies have explored the feasibility of remote monitoring and exercise prescription via PATs, but none have yet published guidelines on the progression of exercise prescription based upon individual fitness as measured by 6MWT or LFI. Without individual tailoring, patients may be given inappropriately low-intensity exercise prescriptions leading to non-progression of physical abilities or worse, further deconditioning. Alternatively, they may be given inappropriately high-intensity exercise prescriptions leading to non-adherence or overtraining causing adverse events. In order to operationalize an exercise prescription using walking as the primary training strategy we explored what would be the thresholds we would need to use by cross-referencing 6MWT and PAT data.

## Methods

A *post hoc* analysis of previously published data was conducted^(3)^. Daily steps from 1-week PAT monitoring data were cross-referenced with baseline 6MWT performance. The latter were categorized in 3 groups: <250 m, 250–349 m, and ≥ 350 m, whereas LFI was used to categorize patients as frail (≥ 4.5), prefrail (3.2–4.4), and robust (< 3.2). Kruskal-Wallis was used for comparisons. Cutoff values were selected based on data distribution (i.e., tertiles) and to align with published literature. Study was approved by the Institutional Review Board and adhered to international regulations (PRO12030073).

## Results

**Figure 1:**
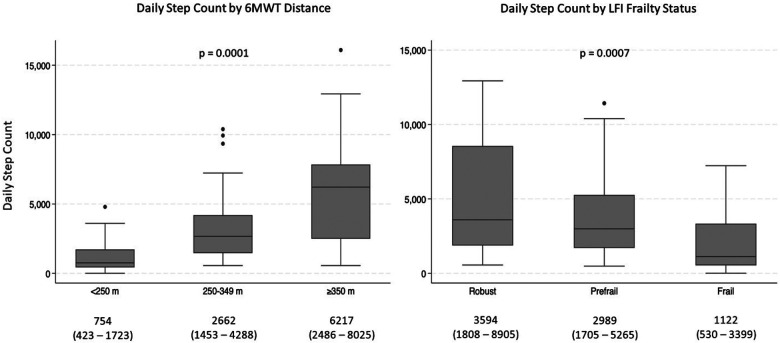
Daily step count according to the 6-minute walk test (6MWT) distance, for participants strolling <250 m, 250–349 m, and ≥350 m to the left; and according to the liver frailty index (LFI) categories, robust, prefrail, and frail, to the right.

In 107 participants with both PAT-daily steps and 6MWT (age 56 ± 11, male 57%), we found that that 19 (18%), 47 (44%) and 41 (38%) accomplished < 250 m, 250–349 m, and ≥ 350 m in their 6MWT, respectively. Participants in the lowest performance category were most likely female (63% vs. 39%), less likely listed for LT (21% vs. 61%), had more ascites (100% vs. 68%) and hepatic encephalopathy (100% vs. 65%), and were most likely frail (79%, vs. 11%). The daily steps per 6WMT category are shown in Fig. 1. Grouping daily steps by LFI category produced similar results (Fig. 1).

## Conclusion

LT candidates are highly sedentary and low daily step counts are associated with increased mortality and hospitalization rates^(3)^, however, exercise interventions are a proven strategy to improve cardiorespiratory fitness, frailty and sarcopenia^(1, 4)^. Since few LT centers have access to a formal prehabilitation program, scalable and generalizable exercise interventions are utterly needed for LT candidates. As an exercise prescription, walking is readily available to most and daily steps are a good surrogate for physical fitness. Thus, a walking prescription considering an increasing daily step count should serve LT programs as a scalable and generalizable prehabilitation strategy.

PATs are a low-cost and scalable method of tracking exercise and have the potential to facilitate remote exercise prescription^(5)^. Some major challenges can arise, particularly the lack of direct observation leading to uncertainty of the suitability of exercise to safely reach physical activity goals. Although regular check-ins with an exercise professional can help tailor daily step counts to the patient’s needs, such contact would eliminate generalizability of the intervention and its operationalization through simple step goals. By establishing a benchmark for daily step counts on the initial exercise prescription that leverages the utility of routine fitness assessments (i.e., 6MWT or LFI), LT centers can confidently and safely prescribe step count goals to be observed, remotely.

The analysis of our data showed that there are clear daily step count differences between groups separated by 6MWT distance and LFI score, reflecting a candidate’s functional status and the need to tailor the exercise prescription based on such status. Considering our findings, we propose a walking prescription of 1500, 3000, and 6000 steps/day for LT candidates with 6MWT distance of < 250 m, 250–349 m, and ≥ 350 m, respectively. For centers obtaining an LFI at baseline, similar steps/day can be prescribed to the frail, prefrail, and robust, respectively. With this modeling, any provider can deliver an initial walking prescription to ambulatory patients in a credible and safe fashion. Daily step counts can then be adjusted up or down, depending on initial performance, in 500 steps/day intervals every two weeks—as needed and until the subject can accomplish the goals corresponding to the next performance category.

Further investigation should consider implementation and efficacy, and its impact on hospitalization and mortality. Additionally, these values are derived from a single week capture of patients wearing a PAT. Further research is needed to understand tolerable, and necessary rates of progression over the periods of 6–12 weeks in frail, pre-frail, and robust groups, and these relationships with health outcomes, in order to fully operationalize remote exercise prescription based upon daily step counts.

## Data Availability

The data that support the findings of this study are available from the corresponding author upon reasonable request. The content is solely the responsibility of the authors and does not necessarily represent the official views of the National Institutes of Health.

## Abbreviations

6MWT: 6-minute walk test
LFI: Liver frailty index
LT: liver transplant
PAT: personal activity tracker(s)
